# Measured glomerular filtration rate (GFR) significantly and rapidly decreases after radical cystectomy for bladder cancer

**DOI:** 10.1038/s41598-020-73191-0

**Published:** 2020-09-30

**Authors:** Mathieu Rouanne, François Gaillard, Matthias E. Meunier, Yanish Soorojebally, Hoang Phan, Hind Slimani-Thevenet, Anne-Sophie Jannot, Yann Neuzillet, Gérard Friedlander, Marc Froissart, Henry Botto, Pascal Houillier, Thierry Lebret, Marie Courbebaisse

**Affiliations:** 1 Department of Urology, Hôpital Foch, Université Paris-Saclay, 40, Rue Worth, 92150 Suresnes, France; 2 UVSQ-Université Paris-Saclay, Paris, France; 3 Department of Physiology, Functional Explorations Unit, Hôpital Européen Georges Pompidou, Paris, France; 4 Department of Biostatistics, Hôpital Européen Georges Pompidou, Paris, France; 5 Department of Nuclear Medicine, Hôpital Européen Georges Pompidou, Paris, France; 6 INSERM U1151-CNRS UMR8253, Paris, France; 7 Université Paris Descartes, Paris, France; 8 Clinical Research Center and Trial Unit, Centre Hospitalier Universitaire Vaudois, Lausanne, Switzerland; 9 INSERM U1138, CNRS ERL8228, Paris, France

**Keywords:** Bladder, Chronic kidney disease, Surgical oncology

## Abstract

Precise determination of glomerular filtration rate (GFR) is essential for the management of patients with muscle-invasive bladder cancer (MIBC). We aim to describe the early evolution of measured GFR (mGFR) after radical cystectomy and urinary diversion (RCUD) and to identify risk factors for GFR decline. GFR measurement using ^51^Cr-EDTA continuous infusion, estimated GFR (eGFR) from five published equations and renal scintigraphy with split renal function determination were performed before and 6 months after RCUD. Chronic Kidney Disease (mGFR < 60 mL/min/1.73 m^2^) and GFR stages were defined according to the KDIGO guidelines using mGFR. Twenty-seven patients (men 85%, median age 65, IQR 59; 68 years) were included. A total of 20 (74%) patients experienced significant mGFR decline at 6 months postoperatively. Median mGFR decreased from 84.1 pre-operatively (IQR 65.3; 97.2) to 69.9 mL/min/1.73 m^2^ (IQR 55.0; 77.9) 6 months after surgery (p < 0.001). Thirteen (48%) patients had a progression to a worse GFR stage. Of the 22 patients without pre-operative CKD, 5 (23%) developed post-operative CKD. Diabetes mellitus was more frequent in patients in the highest tertile of relative mGFR decline (44% vs. 11%, p = 0.02) and platinum-based adjuvant chemotherapy tended to be more frequently used in these patients (44% vs. 17%, p = 0.06). Importantly, pre-operative weight was independently and negatively associated with post-operative mGFR and with mGFR slope in multivariable analyses. In this prospective series, we demonstrated that early and significant mGFR decline occurred after RCUD and perioperative platinum-based chemotherapy, especially in patients with diabetes mellitus and overweight.

## Introduction

Radical cystectomy and urinary diversion (RCUD) is the standard of care for non-metastatic muscle-invasive bladder cancer (MIBC)^1^. Despite perioperative platinum-based chemotherapy, this treatment only provides 5-year survival in about 50% of the patients. The study of chronic kidney disease (CKD) in this population has never been more crucial as bladder cancer is a disease of middle-aged and elderly people^2^. Recently, several studies have reported that preoperative CKD was an independent predictor of cancer mortality after RCUD in patients with MIBC^3–6^. CKD is also particularly concerning since it has been identified as an independent risk factor of death and cardiovascular events in a wide range of populations^7^. Because CKD is common in elderly patients, practicing onco-urologists should pay more attention to renal function assessment in patients with MIBC^8^.

After RCUD, regular follow-up is an imperative need to detect both oncological relapse and functional impairment. In addition to oncological evaluation, life-long monitoring of renal function is a major issue as most of the patients experience renal function impairment after RCUD^9–19^. However, most series on long term renal function outcomes after RCUD that have been published used different methods for glomerular filtration rate (GFR) assessment as a substitute of direct GFR measurement, although the need of directly measuring GFR in these patients has previously been highlighted. Moreover, the early evolution of measured GFR (mGFR) after RCUD has never been prospectively reported. The main aims of the present study were to describe prospectively the evolution of mGFR using a gold standard method before and 6 months after RCUD and to identify risk factors for early-accelerated mGFR decline.

## Methods

### Patient selection and follow-up

**Figure 1:**
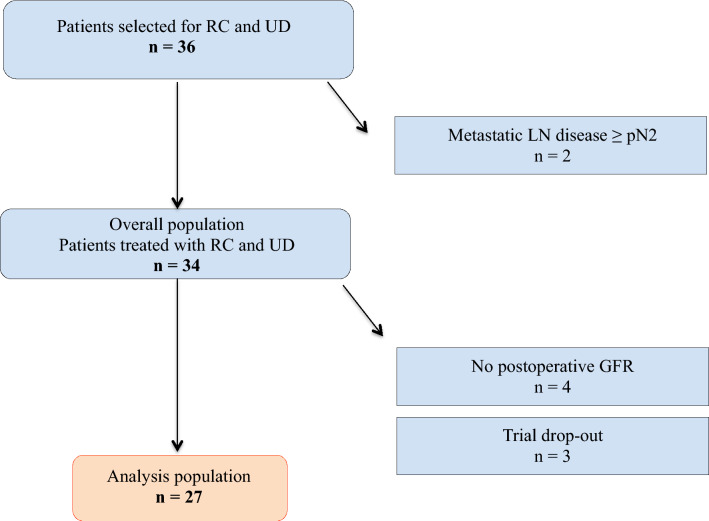
Flow chart of the study. *RC* radical cystectomy, *UD* urinary diversion, *GFR* glomerular filtration rate, *LN* lymph nodes.

Between January 2011 and June 2015, 36 patients selected for RCUD agreed to participate in this study in our institution. This study was conducted in accordance with the Declaration of Helsinki and approved by the French national regulatory board (CNIL Commission Nationale de l’Informatique et des Libertés, N° 915528 and N° 1922081). All patients were informed before any exploration that their data could be used anonymously for clinical research. Of these patients, we excluded 2 patients who did not undergo RCUD due to metastatic lymph nodes involvement (≥ pN2) revealed at extemporaneous examination, 4 patients without postoperative isotopic measurement of GFR and 3 patients who decided to leave the trial, leaving 27 patients available for analysis (Fig. 1). RCUD was performed for clinically localized MIBC (cT2-T4N0M0) in the Urology department, Hôpital Foch, Suresnes, France. Our institutional review board approved the study and written informed consent was obtained from all patients. Clinical, biological and pathological data were collected at baseline. Measured and estimated GFR (mGFR and eGFR, respectively) as well as renal scintigraphy for split renal function determination were simultaneously performed at baseline and at 6 months postoperatively.

### Surgical technique

All patients underwent pelvic lymphadenectomy followed by radical cystectomy. Urinary diversion was achieved using either ileal orthotopic neobladder or ileal conduit. The surgical technique for the ileal orthotopic neobladder has been described previously^20^. In brief, a small bowel segment 45 cm long was resected 20 cm proximal to ileocecal valve. The ileal segment resected was left as found in its natural position without any rotation folding or twist forming a Z. It was detubularized and reconstructed into a low-pressure reservoir. Regarding ileal conduit, a Bricker’s procedure was performed using a 5- to 10-cm long ileal segment, resected 20 cm proximal to ileocaecal valve. The ileal segment was oriented in the isoperistaltic direction and anastomosed to the abdominal wall in a nipple-to stoma fashion. In both cases, the ureters are split and anastomosed separately in the ileal segment or the neobladder. Ureteroileal anastomoses were performed using direct implantation. The ureteral catheters are removed on the 11th or 12th postoperative day. The urethral catheter is removed on the 13th postoperative day.

### GFR measurements

GFR was assessed as previously described through a continuous ^51^Cr-EDTA (GE Healthcare, Little Chalfont, UK) infusion method^21^. A priming dose of 0.5 μCi/kg body weight of ^51^Cr-EDTA was injected intravenously, followed by a constant ^51^Cr-EDTA infusion. After allowing 1 h for equilibration of the tracer in the extracellular fluid, urine was collected and discarded. Average renal ^51^Cr-EDTA clearance was assessed during six consecutive 30-min clearance periods. Blood was drawn at the midpoint of each clearance period with the last collection 300 min after injection of the priming dose. The radioactivity measurements in 1 mL plasma samples and in urine samples were carried out on a Packard Cobra 3 in. crystal γ-ray well counter (PerkinElmer, Waltham, MA, USA).

Theoretical GFR stages were assigned according to the Kidney Disease Improving Global Outcomes (KDIGO) guidelines using mGFR as follows: ≥ 90 mL/min per 1.73 m^2^ for stage 1, 60–89 for stage 2, 45–59 for stage 3A, 30–44 for stage 3B, 15–29 for stage 4, < 15 for stage 5. Faced with the difficulties to properly analyzing the urinary protein/creatinin ratio and hematuria in patients with bladder cancer, we defined CKD as a mGFR < 60 mL/min/1.73 m^2^.

### Creatinine-based estimation of GFR

Estimated GFR (eGFR) was calculated using five equations: Cockcroft-Gault, MDRD, CKD-EPI, Janowitz and FAS equations^22–26^. To allow comparison between these equations and mGFR, eGFR as well as mGFR were all adjusted to body surface area. Both mGFR and eGFR values were obtained simultaneously within a week before surgery and at 6 months postoperative in all 27 patients, at the same institution (Renal physiology department, European Georges Pompidou Hospital, Paris, France). All creatinine measurements were performed in the same laboratory with standardized serum creatinine (SCr) assay using IDMS-traceable enzymatic method. Blood samples were obtained simultaneously with the isotopic GFR measurement.

### Renal scintigraphy and split renal function determination

Renal scintigraphy was performed as previously described in the supine position, with the back of the patient against a wide-field view γ camera (Infinia hawk eye 4, GE) that was equipped with a low-energy, high-resolution all-purpose collimator, which allowed visualization of the kidneys and the heart^27^. The 10% window was centered on the ^99m^Tc 140 keV photopeak, and 200 to 300 MBq of ^99m^Tc-DTPA were injected 30 min after oral hydration (7 mL/kg), during the equilibration or distribution period for clearance determinations. The study included a flow study of 60 frames (128 × 128 pixels) of 1 s each followed by a sequence of 120 frames of 10 s each for 20 min. Split Renal Function (SRF; %) was determined by the Patlak–Rutland method, with both extravascular and intravascular background corrections, with the use of subrenal and splenic background regions of interest (ROI), respectively. The ROI of both kidneys were determined on a summed frame (from 1 min 30 s to 2 min 30 s). Depth attenuation was corrected with the use of lateral views to determine the skin-to-kidney center distance and a linear attenuation coefficient of 0.12 cm^−1^ for ^99m^Tc in soft tissues. On the basis of nuclear renography, we defined renal asymmetry as an SRF superior or equal to 45/55. A change in SRF was considered as significant if it was > 5%.

### Statistical analysis

Continuous, normally distributed and non-normal distributed variables are reported in median and interquartile range (IQR: 25p–75p). Categorical variables are reported in number and percentage. Paired *t* tests were used for perioperative comparison of renal function change. Median residual was defined as the difference between mGFR and eGFR, and assessed at baseline and at 6 months postoperatively for all patients. All tests were two sided, and a *p* value < 0.05 was considered statistically significant. Statistical analyses were performed using the SPSS software (SPSS Inc, Chicago, IL, USA) and R [R Core Team (2019)]. R: A language and environment for statistical computing and R (Foundation for Statistical Computing, Vienna, Austria). Pearson correlation coefficients were calculated for the association between eGFR and mGFR. Univariate comparisons were conducted between the group of patient who developed CKD stage 3 or 4 and the group of patient who did not. The slope of mGFR was calculated for unindexed mGFR (i.e. expressed in mL/min and not mL/min/1.73 m^2^) because indexation is not necessary for intra-individual comparisons before and after surgery. We designed two multivariable linear regression models for mGFR slope (before-after surgery) and postoperative mGFR. Variables were selected by exhaustive search (including, forward, backward and sequential replacement) to maximize the adjusted R-squared and minimize the Bayesian Information Criteria. A maximum of two variables was allowed in each model, due to the number of patients included in the study. In order to find the two best variables for our models, all the following variables including age, weight, BMI, height, body surface area, creatinine, estimated GFR, measured GFR, proteinuria, albuminuria, creatininuria, urinary creatinine clearance, urinary sodium, urinary potassium, and urinary urea have been screened according to an exhaustive search.

## Results

### Patients characteristics

The clinical, biological and pathological features of the entire cohort at baseline (n = 34) are reported in Table 1. For the 27 patients included in the analysis (men 85%), median age was 65 years (IQR 59; 68). At baseline, 22 (81%) patients had a mGFR superior to 60 mL/min/1.73 m^2^ and 5 (19%) patients had a mGFR between 30 and 59 mL/min/1.73 m^2^ and consequently a CKD. A total of 20 (74%) and 7 (26%) patients underwent ileal orthotopic neobladder and ileal conduit urinary diversion respectively. All the patients were managed in conventional hospital ward and no transfer into intensive care unit was noticed. While 1 (4%) patient received intraoperative blood transfusion, we did not observe any other intraoperative events such as severe hypotension that could have impacted immediate kidney function. Among the patients cohort, 24 (89%) patients had their catheters removed at day 13–14, and 3 (11%) patients had their catheters removed at day 15–17. Overall, 8 (30%) patients received neoadjuvant cisplatin-based chemotherapy and 7 (26%) patients were treated with adjuvant chemotherapy. Additionally, 2 out of 27 (4%) patients received systemic aminoglycosides during 3 days for postoperative acute pyelonephritis. Nonsteroidal anti-inflammatory drugs have not been used in the postoperative time period.

**Table 1:** Patients characteristics at baseline for overall population, excluded population, and analysis population at baseline and 6 months postoperatively.

	Overall population (n = 34)	Analysis population (n = 27)	Excluded population (n = 7)
Age, years (range)	66 [60.2; 68.8]	65 [59; 68]	66 [63; 78]
**Characteristics at baseline, No. (%)**			
**Sex**			
Male	30 (88)	23 (85)	7(100)
Female	4 (12)	4 (15)	0 (0)
Hypertension	25 (74)	20 (74)	5 (71)
Diabetes	8 (24)	6 (22)	2 (29)
**Smoking habits**	28 (82)	23 (85)	5 (71)
Non-smokers	6 (18)	4 (15)	2(29)
Past- history of smoking	11 (32)	10 (37)	1 (14)
Active smokers	17 (50)	13 (48)	4 (57)
BMI, kg/m^2^	28.3 [25.5; 30.0]	28.1 [25.4; 29.8]	30.0 [27.3; 31.1]
Serum creatinine, µmol/L	66.0 [56.2; 82.5]	64 [55; 83]	68 [57; 85]
mGFR, mL/min/1.73 m^2^	82.2 [65.9; 96.3]	84.1 [65.3; 97.2]	78.0 [66.8; 96.0]
**KDIGO GFR staging**			
G1 (≥ 90 mL/min/1.73 m^2^)	12	9	3
G2 (60–89 mL/min/1.73 m^2^)	16	13	3
G3a (45–59 mL/min/1.73 m^2^)	2	1	1
G3b (30–44 mL/min/1.73 m^2^)	4	4	0
G4 (15–29 mL/min/1.73 m^2^)	0	0	0
G5 (< 15 mL/min/1.73 m^2^)	0	0	0
**Urinary diversion**			
Ileal orthotopic neobladder	24 (71)	20 (74)	4 (57)
Ileal conduit urinary diversion	10 (29)	7 (26)	3 (43)
**Pathological stage**			
≤ pT2	15 (44)	14 (52)	1 (14)
pT3-T4	19 (56)	13 (48)	6 (86)
pN+	10 (29)	6 (22)	4 (57)
**Perioperative chemotherapy**	–	15 (56)	–
Neoadjuvant/induction (MVACx4 or GCx4)	–	8 (29)	–
Adjuvant (MVACx4)	–	7 (26)	–
**Characteristics at 6 mo. postoperatively No. (%)**			
Hydronephrosis	–	5 (19)	–
Urinary tract infection^a^	–	8 (30)	–
Renal asymmetry	–	8 (30)	–

Continuous variables: median [IQR]; categorical variables: n (%).

*BMI* body mass index, *mGFR* measured glomerular filtration rate, *KDIGO* Kidney Disease, *MVAC* Methothrexate-Vinblastine-Adriamycin-Cisplatin, *GC* Gemcitabine-Cisplatin.

^a^Documented by bacteriological urine analysis.

### Variation of glomerular filtration rate and of chronic kidney disease stages

**Figure 2:**
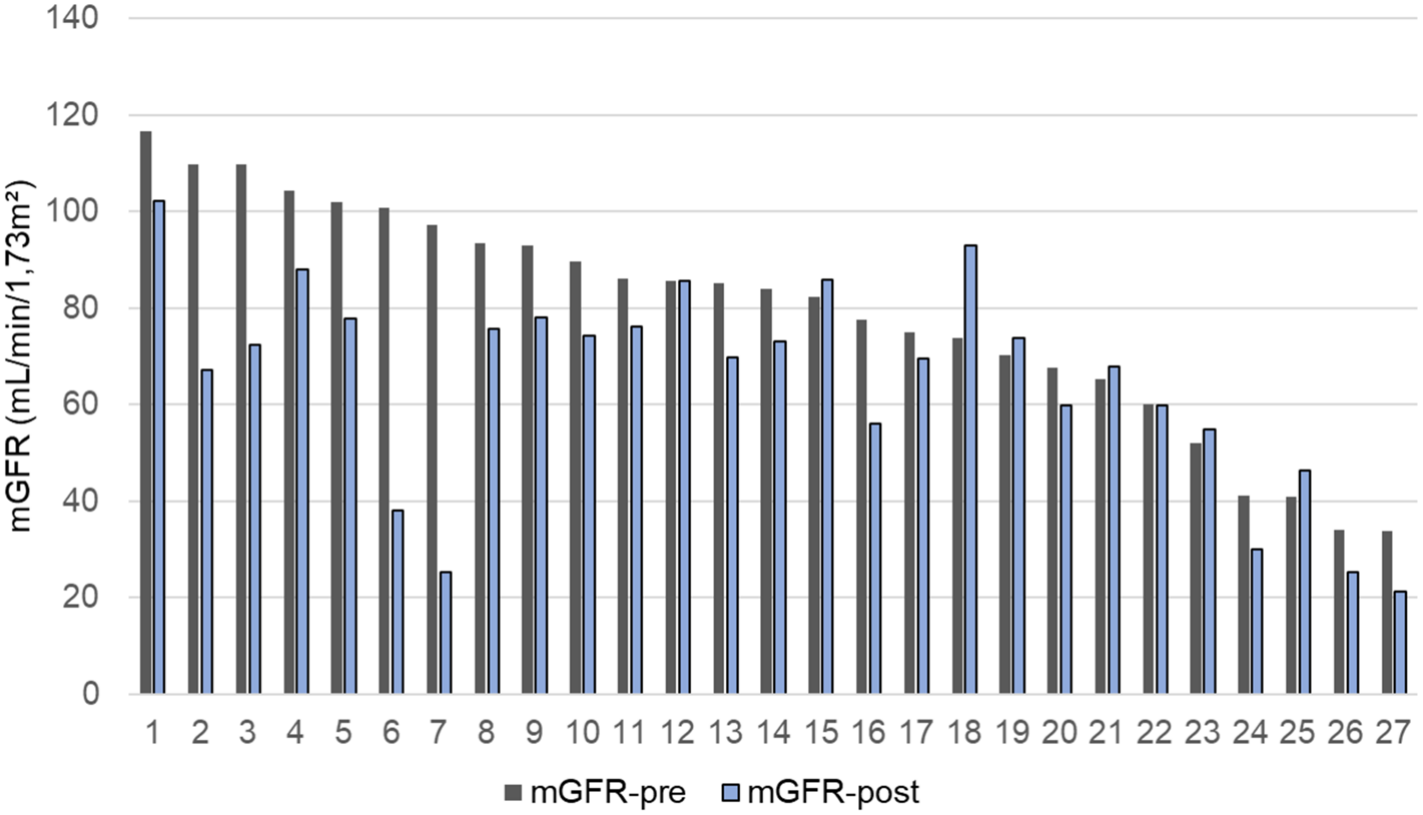
Individual evolution of measured glomerular filtration rate (mGFR in mL/min/1.73 m^2^) before and 6 months after radical cystectomy and urinary diversion. Black bars: mGFR before surgery. Grey bars: mGFR after surgery. Dotted lines represents mGFR at 60 mL/min/1.73 m^2^ (cut-off for chronic kidney disease definition) and at 30 mL/min/1.73 m^2^ (cut-off between stages 3B and 4 for GFR staging).

A total of 20 (74%) patients experienced postoperative mGFR decline at 6 months among them, 3 (11%) patients experienced a postoperative eGFR increase, including the two patients treated with aminoglycoside antibiotics, highlighting the discrepancy between creatinine measure and GFR measurement. The overall discordance rate for GFR variation between eGFR and mGFR was 18.5%, highlighting the need for mGFR before and after surgery. The median mGFR decreased from 8.1 mL/min/1.73 m^2^ preoperatively (IQR 65.3; 97.2) to 69.9 mL/min/1.73 m^2^ (IQR 55.0; 77.9) 6 months after surgery (p < 0.001). We also found a significant decrease in median eGFR between baseline and follow-up according to Cockroft-Gault (p = 0.002), MDRD (p = 0.004), CKD-EPI (p = 0.003), Janowitz (p = 0.003) and FAS equations (p = 0.003). These results are presented in Supplementary Information (Supplementary Table S1). Last, we present for each patient the correspondence between eGFR and mGFR variations in Supplementary Fig. S1. According to preoperative and postoperative mGFR values, 13 (48%) patients developed a worse GFR stage after RCUD. Of the 22 patients without CKD (pre-operative mGFR ≥ 60 mL/min/1.73 m^2^) before surgery, 11 (50%) had a progression in GFR stages (6 from stage 1 to stage 2, 3 from stage 2 to stage 3A, 1 from stage 1 to stage 3B and 1 from stage 1 to stage 4) and 5 patients (23%) developed post-operative CKD (mGFR < 60 mL/min/1.73 m^2^). Of the 5 patients with initial CKD, 2 (40%) had a progression in GFR stages (from stage 3B to 4). Figure 2 represents the individual evolution of mGFR before and 6 months after RCUD.

### Variation of split renal function and of renal asymmetry

All patients underwent scintigraphy, both preoperatively and postoperatively. Preoperatively, 10 patients (37%) had a renal asymmetry and six months after surgery, 13 patients (48%) had a renal asymmetry. In details, between pre and postoperative evaluation, 5 patients (18.5%) developed a new renal asymmetry and pre-operative renal asymmetry disappeared in 2 patients (7.4%). Of note the 5 patients who developed a new renal asymmetry, 2 did so by an increase ≤ 5% of the SRF, and the 2 patients who switched from asymmetry to equivalent renal function did so by a decrease ≤ 5% of the SRF, which is a modification that cannot be considered as significant. In total, between preoperative and postoperative evaluation, 3 patients (11.1%) developed a new renal asymmetry by an increase > 5% of the SRF.

### Variables associated with glomerular filtration rate and chronic kidney disease stages variation

No variable was found to be different between patients developing CKD stage 3 (n = 5) and those not developing CKD stage 3 (n = 22) (Supplementary Table S2). On the contrary, we observed that patients who developed CKD stage 4 (n = 3) were more frequently diabetic (100% vs 12.5%, p = 0.006), and had a higher pre-operative weight (119.8 ± 26.2 kg vs. 76.9 ± 14.4 kg, p < 0.0001). They also had a lower preoperative mGFR (55 ± 36.5 mL/min/1.73 m^2^ vs. 81.9 ± 20.7 mL/min/1.73 m^2^; p = 0.04), a higher urinary proteinuria (863.3 ± 500.1 mg/L vs. 200.8 ± 224.4 mg/L; p < 0.0001) and a higher albuminuria (449 ± 348.5 mg/L vs. 70 ± 81.1 mg/L; p < 0.0001) than those who did not develop CKD stage 4 (Supplementary Table S2).

We compared the characteristics of patients between the tertile with the highest percentage of mGFR decline and the two lower tertiles. Results are summarized in Table 2. The proportion of patients with diabetes mellitus was significantly higher in the subgroup of patients in the highest tertile of relative mGFR decline (44% vs. 11%, p = 0.02). Platinum-based adjuvant chemotherapy tended to be more frequently used in these patients (44% vs. 17%, p = 0.06). Post-surgery hydronephrosis also tended to be more frequent in the patients with the highest relative GFR decline (33% vs. 11%, p = 0.08). Neither baseline renal asymmetry nor incident renal asymmetry occurring after RCUD were associated with an accelerated mGFR decline.

**Table 2:** Comparison of patients’ characteristics in the highest tertile of relative mGFR decline versus the two lower tertiles.

	Low and middle tertiles of relative mGFR decline	High tertile of relative mGFR decline	p-value
	n = 18	n = 9	
**Baseline characteristics**			
Gender (M)	78	100	0.06
Age at surgery (years)	66.2 (7.26)	63.0 (8.86)	0.73
BMI (kg/m^2^)	26.7 (3.51)	31.6 (10.9)	0.22
Hypertension (%)	78	67	0.73
Diabetes mellitus (%)	11	44	0.02
Past or current smokers (%)	78	100	0.06
mGFR (mL/min/1.73 m^2^)	79.2 (18.4)	78.5 (33.0)	0.95
Renal asymmetry (%)	39	33	0.61
**Treatment characteristics and post- surgery complications**			
**Urinary diversion**			
Ileal orthotopic neobladder (%)	72	78	0.38
Ileal conduit urinary diversion (%)	23	22	0.18
**Perioperative chemotherapy**^a^	50	67	0.21
Induction	11	0	0.85
Neoadjuvant	22	22	0.5
Adjuvant	17	44	0.06
Urinary tract infection	28	33	0.38
Hydronephrosis	11	33	0.08
**Post-surgery renal asymmetry**			
Incident renal asymmetry^b^	5.6	22.2	0.13

Results are shown as percentage for qualitative data or mean (standard deviation) for quantitative data.

*M* male, *BMI* body mass index, *mGFR* measured glomerular filtration rate.

^a^All chemotherapy treatments included platinum salts.

^b^Incident renal asymmetry was defined as the occurrence of a significant renal asymmetry after surgery (split renal function superior or equal to 45/55) due to a change in the split renal function > 5%.

In Supplementary Table S3, we present univariate analysis for pre-operative variables associated with post-operative mGFR and mGFR slope, respectively. In Tables 3 and 4, we present multivariable models for post-operative mGFR and mGFR slope, respectively. We observed that pre-operative mGFR was independently and positively associated with post-operative mGFR whereas it was independently and negatively associated with mGFR slope (meaning that if the pre operative mGFR is higher, the absolute decrease of mGFR after surgery will be higher). Of note, we observed that pre-operative weight was independently and negatively associated with post-operative mGFR and with mGFR slope (meaning that if the pre operative weight is higher, the absolute decrease of mGFR after surgery will be higher).

**Table 3:** Multivariable model summary for post-operative mGFR.

	Estimate	95% CI	p-value
Weight	− 0.39	− 0.74; − 0.03	0.01
mGFR	0.40	0.09; 0.71	0.03

For this model, intercept was 64.8 [18.9–110.6] and adjusted R^2^ was 0.44.

**Table 4:** Multivariable model summary for mGFR slope.

	Estimate	95% CI	p-value
Weight	− 0.55	− 0.98; − 0.13	0.01
mGFR	− 0.68	− 1.05; − 0.31	< 0.001

For this model, intercept was 82.2 [27.4–137.0] and adjusted R^2^ was 0.34.

### Concordance rates between eGFR and mGFR for GFR stages

**Figure 3:**
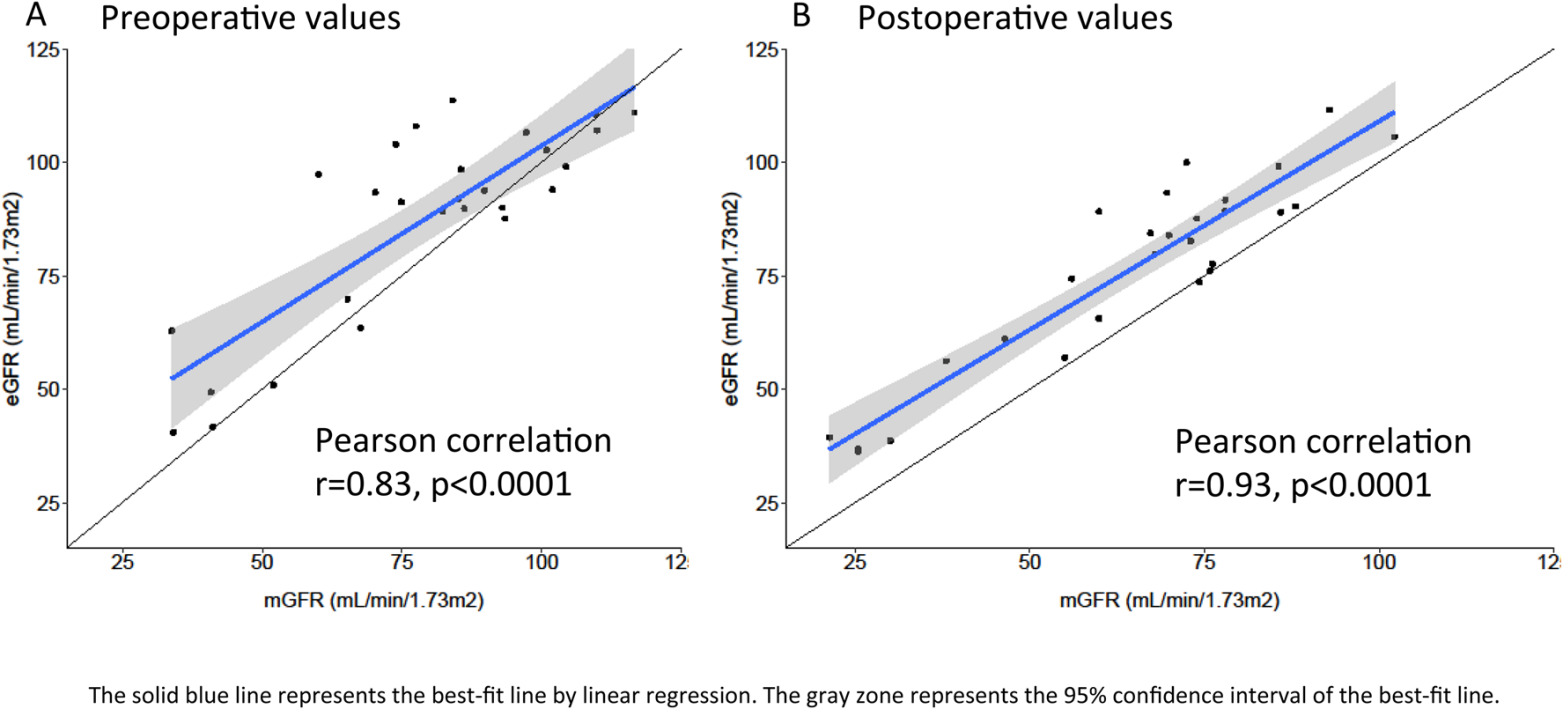
Scatter plot showing the relationship between eGFR and mGFR values ((**A**) preoperative values; (**B**) postoperative values).

The relationship between mGFR and eGFR values is represented by a scatter plot in Fig. 3. The Pearson correlation coefficient between eGFR and mGFR was r = 0.83, p < 0.0001 for pre-operative values and r = 0.93, p < 0.0001 for post-operative values. The concordance rate between eGFR (estimated with the CKDEPI equation) and mGFR was 56% preoperatively and 52% postoperatively. Similar results were obtained with the MDRD equation (59% preoperatively and 52% postoperatively). Concordance rate for KDIGO GFR stages preoperatively and postoperatively between eGFR using the 5 equations and mGFR are presented in Table 5. Residuals between mGFR and eGFR are presented in Supplementary information (Supplementary Table S4).

**Table 5:** Concordance rate for KDIGO GFR staging between measured GFR and estimated GFR.

**Preoperative concordance rate for KDIGO GFR staging**	
BSA adjusted Cockcroft-Gault	44%
MDRD	59%
CKD-EPI	56%
BSA adjusted Janowitz	56%
FAS	63%
**Postoperative concordance rate for KDIGO GFR staging**	
BSA adjusted Cockcroft-Gault	59%
MDRD	52%
CKD-EPI	52%
BSA adjusted Janowitz	56%
FAS	70%

*BSA* body surface area.

## Discussion

In the present study, we prospectively investigated the evolution of mGFR with a gold standard method for GFR measurement using ^51^Cr-EDTA continuous infusion. First, we identified for the first time an early decline in mGFR after RCUD and perioperative platinum-based chemotherapy with half of the patients developing a worse mGFR stage only 6 months after surgery, excluding a significant contribution of aging to explain this GFR decline. Specifically, renal function assessment based on estimated GFR values could delay the diagnosis and de facto the management of chronic kidney disease. In that regard, physicians should consider gold standard method for GFR measurement in the follow-up of these patients. Overall, these data support the crucial need to carefully monitor GFR before and after RCUD. Second, we identified preoperative weight and mGFR as independent factors associated with post-operative mGFR and mGFR slope. RCUD is the gold standard treatment for patients with MIBC, but this procedure is associated with significant risks of morbidity and mortality, notably due to renal function impairment. Despite the variety of urinary diversion techniques, bladder cancer patients treated with radical cystectomy face a life-long risk of CKD^10^. Additionally, preoperative CKD in patients with urothelial carcinoma has been significantly associated with unfavorable clinical outcome^3,5^. Therefore, accurate evaluation of GFR before surgery and of GFR evolution over time is a paramount of importance in patients with ileal interposed on their urinary tract. Measurement of GFR with a gold standard method has never been performed prospectively in patients with MIBC, before and after RCUD^16^. Deteriorating renal function was expected in patients treated with RCUD, but not in such a short delay^12,28^. Available studies are retrospective and mainly based on GFR estimation, with only one study with both measured GFR and estimated GFR using the MDRD equation. All these studies demonstrated a decline of renal function after 5–10 years of follow-up. Risk factors for GFR decline include age, nephrotoxicity of chemotherapy or medications chronic hypertension, diabetes mellitus, and potentially modifiable factors such as mechanical obstruction or acute urinary infection^9,13,19^. Interestingly, we found that the proportion of diabetic patients was more important in the tertile of patients with the highest relative decline in mGFR and that patients who developed CKD stage 4 after surgery were more frequently diabetic. That is not surprising, as diabetes is in itself a risk factor for CKD. However, this finding highlights the need to pay a special attention to early GFR decline in patients with diabetes mellitus after RCUD. Notably, we also found that patients who developed CKD stage 4 had a higher pre-operative weight and that pre-operative weight was independently and negatively associated with post-operative mGFR and with mGFR slope (meaning that if the pre operative weight is higher, the absolute decrease of mGFR after surgery will be higher). These results suggest that overweight should also be considered as a risk factor for pejorative GFR evolution after RCUD. This finding is potentially related to the fact that overweight is itself associated with a state of glomerular hyperfiltration, with a lower renal reserve resulting in a poorer renal compensatory response after kidney injury^29,30^. This is in line with our finding that pre-operative mGFR is independently and negatively associated with mGFR slope (meaning that if the pre operative mGFR is higher, the absolute decrease of mGFR after surgery will also be higher).

The high proportion of deteriorating renal function after RCUD in our series could be explained by the fact that over half of our patients (56%) underwent peri-operative platinum-based chemotherapy, which is much more than in other published cohorts. This hypothesis is supported by the trend to a higher proportion of patients with adjuvant chemotherapy in the tertile of patients with the highest relative mGFR decline in our study^9,13^. Although delayed nephrotoxicity, post neoadjuvant chemotherapy may also result in postoperative mGFR decline, this association could not be properly assessed due to the timing of mGFR after neoadjuvant therapy. Of note, it has been reported that cisplatin based-neoadjuvant chemotherapy was an independent predictor of worsening of at least one MDRD-eGFR stage up to 12 months after RCUD^31^.

In order to study the occurrence of renal asymmetry after surgery and the impact of pre and post-operative renal asymmetry on mGFR, we performed a renal scintigraphy to assess SRF for all patients at baseline and 6 months after surgery. Although more than one third of our patients had significant renal asymmetry before surgery, the incidence of renal asymmetry after RCDU was low (11% of our patients) and neither baseline renal asymmetry nor renal asymmetry occurring after RCUD were associated with an accelerated mGFR decline. These results suggest that SRF assessment with renal scintigraphy should not be systematically performed to predict post-surgery mGFR evolution for patients with MIBC undergoing RCUD.

It was reported that GFR estimation using the MDRD formula was unreliable in detecting renal function loss during follow-up after RCUD, highlighting the need to measure GFR in these patients. Appropriate GFR staging is necessary to adapt treatments dosage and patient’s follow-up^32^.

The present study has several limitations. First, the number of patients included in our protocol study represented approximately 10% of the workflow of patients who underwent a radical cystectomy in our department within this time period. Thus, the sample size of our cohort study may induce a potential selection bias. Arguably, the complexity and time-consuming nature of isotopic GFR measure has prevented the inclusion of more patients. Second, our results are monocentric and would have ideally required an external validation with a larger cohort. Additionally, the limited number of patients included in our study did not allow the appropriate comparison between the five equations regarding their performances against mGFR (percentage within 10%, percentage within 30%, correlation, concordance). Aware of this, we focused our analyses on the concordance between eGFR and mGFR for GFR staging. However, in the setting of MIBC, the question is not to reach a 1 mL/min precision of GFR but to correctly classify patients according to their GFR stages in order to adapt treatments dosage and follow-up. Overall our results are in favor of GFR measurement using a gold standard method in these patients. Finally, longer follow-up with repeated GFR measurements would be needed to confirm if this decrease in mGFR is only initial or extended in time.

A major strength of this study is the prospective design and the use of gold standard technique that allow us to precisely quantify the early loss of renal function after RCUD. Use of mGFR avoided the confounding factor of denutrition and changes in body composition, that are frequent in patients with cancer, and significantly impair interpretation of eGFR^33^. Importantly, use of mGFR probably contributed to unravel a surprisingly early loss of renal function, setting RCUD as a high-risk condition for CKD.

## Conclusions

In our prospective series, we demonstrated a significant and early decrease in mGFR after RCUD and perioperative platinum-based chemotherapy in the majority of our patient cohort (74%), especially in patients with diabetes mellitus and overweight. Because early GFR impairment is frequent in this population, we highlight the importance to carefully monitor GFR before and after RCUD using a gold standard method for GFR measurement. These findings may help urologists and oncologists to harmonize renal function assessment in patients with MIBC.

## Supplementary information

Supplementary Information.
