# Author Correction: Measured glomerular filtration rate (GFR) significantly and rapidly decreases after radical cystectomy for bladder cancer

**DOI:** 10.1038/s41598-021-85466-1

**Published:** 2021-03-11

**Authors:** Mathieu Rouanne, François Gaillard, Matthias E. Meunier, Yanish Soorojebally, Hoang Phan, Hind Slimani-Thevenet, Anne-Sophie Jannot, Yann Neuzillet, Gérard Friedlander, Marc Froissart, Henry Botto, Pascal Houillier, Thierry Lebret, Marie Courbebaisse

**Affiliations:** 1 Department of Urology, Hôpital Foch, Université Paris-Saclay, 40, Rue Worth, 92150 Suresnes, France; 2 UVSQ-Université Paris-Saclay, Paris, France; 3 Department of Physiology, Functional Explorations Unit, Hôpital Européen Georges Pompidou, Paris, France; 4 Department of Biostatistics, Hôpital Européen Georges Pompidou, Paris, France; 5 Department of Nuclear Medicine, Hôpital Européen Georges Pompidou, Paris, France; 6 INSERM U1151-CNRS UMR8253, Paris, France; 7 Université Paris Descartes, Paris, France; 8 Clinical Research Center and Trial Unit, Centre Hospitalier Universitaire Vaudois, Lausanne, Switzerland; 9 INSERM U1138, CNRS ERL8228, Paris, France

Correction to: *Scientific Reports* https://doi.org/10.1038/s41598-020-73191-0, published online 30 September 2020

The original version of this Article contained a typographical error in the spelling of the author Matthias E. Meunier, which was incorrectly given as Matthias Meunier. This has now been corrected in the PDF and HTML versions of the Article, and in the accompanying Supplementary Information file.

