# Supplementary material for: Measured glomerular filtration rate (GFR) significantly and rapidly decreases after radical cystectomy for bladder cancer
Source: Sci Rep. 2020 Sep 30;10:16145. doi: 10.1038/s41598-020-73191-0 (PMC7528003; doi:10.1038/s41598-020-73191-0)
Supplement: Supplementary file 1 — Supplementary Information. [file 41598_2020_73191_MOESM1_ESM.docx]

**Supplementary Information**

**Measured Glomerular Filtration Rate (GFR) Significantly and Rapidly Decreases After Radical Cystectomy for Bladder Cancer**

Mathieu Rouanne^1,2*^, François Gaillard^3¥^, Matthias E.Meunier^1¥^, Yanish Soorojebally^1,2^, Hoang Phan^4^, Hind Slimani-Thevenet^5^, Anne-Sophie Jannot^4^, Yann Neuzillet^1,2^, Gérard Friedlander^3,6,7^, Marc Froissart^8^, Henry Botto^1^, Pascal Houillier^3,6,9^, Thierry Lebret^1,2^, Marie Courbebaisse^3,5,6^

¥These two authors contributed equally to this work.

*1. Department of Urology, Hôpital Foch, Suresnes, France*

*2. Université Versailles-Saint-Quentin-en-Yvelines, Université Paris-Saclay, France*

*3. Department of Physiology, Functional Explorations Unit, Hôpital Européen Georges Pompidou, Paris, France*

*4. Department of Biostatistics, Hôpital Européen Georges Pompidou, Paris, France*

*5. Department of Nuclear Medicine, Hôpital Européen Georges Pompidou, Paris, France*

*6. INSERM U1151-CNRS UMR8253, Paris, France,*

*7. Université Paris Descartes, Paris, France*

*8. Clinical Research Center and Trial Unit, Centre Hospitalier Universitaire Vaudois, Lausanne, Switzerland*

*9. INSERM U1138, CNRS ERL8228, Paris, France.*

**Figure S1. Correspondence between mGFR and eGFR variations at the individual level**

**
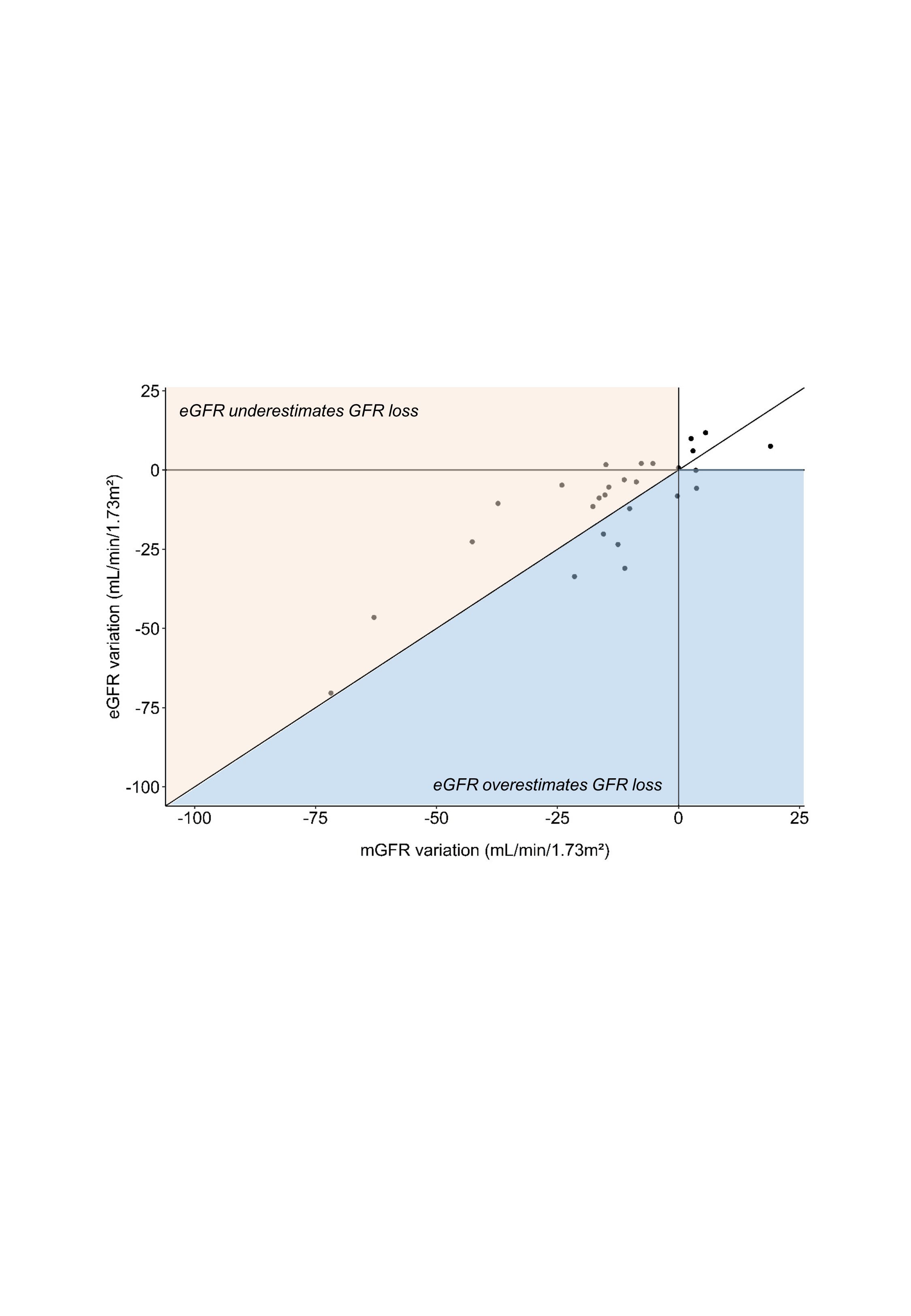
**

**Table S1. Comparison of preoperative and postoperative renal function**

|  | **Preoperative**  *Mean [IC95%]* | **Postoperative**  *Mean [IC95%]* | **Mean difference**  *Mean [IC95%]* | ***p* value *** |
| --- | --- | --- | --- | --- |
| **Measured GFR,**  **mL/min/1.73 m^2^** | 79.0  [69.6 ; 88.3] | 64.7  [56.2; 73.3] | -14.2  [-22.2; -6.3] | 0.001 |
| **Estimated GFR,**  **mL/min/1.73 m^2^** |  |  |  |  |

| **BSA adjusted Cockcroft-Gault** | 93.3  [82.4 ; 104.2] | 76.3  [67.4 ; 85.3] | -17.0  [-27.7 ; -6.3] | 0.003 |
| --- | --- | --- | --- | --- |
| **MDRD** | 96.4  [83.9 ; 109.0] | 79.4  [68.2 ; 90.7] | -17.0  [-28.3 ; -5.7] | 0.005 |
| **CKD-EPI** | 87.4  [78.7 ; 96.1] | 76.7  [68.3 ; 85.2] | -10.7  [-17.9 ; -3.4] | 0.005 |

| **BSA adjusted Janowitz** | 84.2  [78.9 ; 89.5] | 76.9  [72.1 ; 81.7] | -7.3  [-12.0 ; -2.6] | 0.004 |
| --- | --- | --- | --- | --- |
| **FAS** | 89.1  [78.1 ; 100.2] | 74.4  [65.0 ; 83.9] | -14.7  [-24.2 ; -5.2] | 0.004 |

* Calculated with Paired t-test, GFR: glomerular filtration rate, BSA: body surface area

**Table S2.** Comparison of preoperative characteristics between patients who developed CKD stage 3 (Table S2A) or stage 4 (Table S2B) and those who did not. Data are presented as percentage for categorical variables or mean and SD.

*Table S2A*

| Variable | No CKD3 (n=22) | CKD3  (n=5) | p-value |
| --- | --- | --- | --- |
| Female | 2 (9.1) | 2 (40.0) | 0.28 |
| No hypertension | 6 (27.3) | 1 (20.0) | 1.00 |
| No diabetes | 18 (81.8) | 3 (60.0) | 0.64 |
| Non smokers | 2 (9.1) | 2 (40.0) | 0.28 |
| No Infection | 16 (72.7) | 3 (60.0) | 0.98 |
| No Hydronephrosis_drain | 19 (86.4) | 3 (60.0) | 0.46 |
| Age (years) | 65.5 (7.1) | 63 (11.3) | 0.52 |
| Weight (kg) | 82.9 (21.4) | 76.3 (17.8) | 0.52 |
| BSA (m²) | 1.9 (0.2) | 1.8 (0.3) | 0.46 |
| Creatinine (µmol/L) | 80.3 (33.1) | 62.6 (8.7) | 0.24 |
| eGFR (mL/min/1.73m²) | 85.5 (22.6) | 95.7 (18.4) | 0.35 |
| mGFR (mL/min/1.73m²) | 78.6 (25) | 80.7 (17.9) | 0.86 |
| Proteinuria (mg/L) | 303.6 (357.1) | 146 (110.6) | 0.33 |
| Albuminuria (mg/L) | 127.9 (187.6) | 42.8 (45.8) | 0.32 |
| Stage (pT) |  |  |  |
| ≤1 | 8 (36.3) | 1 (20.0) | 0.98 |
| 2 | 4 (18.2) | 1 (20.0) |  |
| 3a | 2 (9.1) | 1 (20.0) |  |
| 3b | 4 (18.2) | 1 (20.0) |  |
| 4a | 4 (18.2) | 1 (20.0) |  |
| Stage (pN) |  |  |  |
| 0 | 18 (81.8) | 3 (60.0) | 0.19 |
| 1 | 2 (9.1) | 0 (0.0) |  |
| 2 | 2 (9.1) | 2 (40.0) |  |

BSA: Body surface area, eGFR: estimated GFR with the CKD EPI equation, mGFR: measured GFR,

*Table S2B*

| Variable | No CKD 4 (n=24) | CKD 4 (n=3) | p-value |
| --- | --- | --- | --- |
| Female | 4 (16.7) | 0 (0.0) | 1.00 |
| No hypertension | 7 (29.2) | 0 (0.0) | 0.69 |
| No diabetes | 21 (87.5) | 0 (0.0) | 0.006 |
| No Tobacco | 4 (16.7) | 0 (0.0) | 1.00 |
| No Infection | 17 (70.8) | 2 (66.7) | 1.00 |
| No Hydronephrosis_drain | 20 (83.3) | 2 (66.7) | 1.00 |
| Age (years) | 65.1 (8.3) | 64.7 (1.5) | 0.93 |
| Weight (kg) | 76.9 (14.4) | 119.8 (26.2) | < 1e-04 |
| BSA (m²) | 1.9 (0.2) | 2.2 (0.2) | 0.003 |
| Creatinine (µmol/L) | 73.6 (27) | 104.3 (51.5) | 0.09 |
| eGFR (mL/min/1.73m²) | 89.6 (20.1) | 70.1 (33.6) | 0.13 |
| mGFR (mL/min/1.73m²) | 81.9 (20.7) | 55 (36.5) | 0.04 |
| Proteinuria (mg/L) | 200.8 (224.4) | 863.3 (500.1) | < 1e-04 |
| Stage (pT) |  |  |  |
| ≤1 | 8 (33.3) | 1 (33.3) | 0.88 |
| 2 | 4 (16.7) | 1 (33.3) |  |
| 3 | 7 (29.2) | 1 (33.3) |  |
| 4a | 5 (20.8) | 0 (0.0) |  |
| Stage (pN) |  |  |  |
| 0 | 19 (79.2) | 2 (66.7) | 0.58 |
| 1 | 2 (8.3) | 0 (0.0) |  |
| 2 | 3 (12.5) | 1 (33.3) |  |

BSA: Body surface area, eGFR: estimated GFR with the CKD EPI equation, mGFR: measured GFR,

**Table S3.** Univariate analysis for mGFR slope (Table S3A) and post-operative mGFR respectively (Table S3B). UCrCl, urinary creatinine clearance. Uu Urinary urea, Uk: urinary potassium, Una : urinary sodium

*Table S3A*

|  | r | P-value |
| --- | --- | --- |
| Age (years) | 0,11 | 0,57 |
| Weight (kg) | -0,18 | 0,37 |
| BMI (kg/m²) | -0,10 | 0,62 |
| Height (cm) | -0,22 | 0,27 |
| BSA (m²) | -0,23 | 0,24 |
| Creatinine (µmol/L) | 0,25 | 0,21 |
| eGFR (mL/min/1.73m²) | -0,33 | 0,10 |
| mGFR (mL/min/1.73m²) | -0,46 | 0,02 |
| Proteinuria (g/L) | -0,00 | 0,99 |
| Albuminuria (g/L) | -0,01 | 0,96 |
| Creatininuria (mmol/L) | -0,07 | 0,73 |
| UCrCl (mL/min) | -0,45 | 0,02 |
| UNa (mmol/L) | -0,26 | 0,20 |
| Uk (mmol/L) | -0,20 | 0,33 |
| Uu (mmol/L) | -0,42 | 0,03 |

*Table S3B*

|  | r | P-value |
| --- | --- | --- |
| Age (years) | 0,08 | 0,70 |
| Weight (kg) | -0,57 | 0,00 |
| BMI (kg/m²) | -0,54 | 0,00 |
| Height (cm) | -0,16 | 0,43 |
| BSA (m²) | -0,51 | 0,01 |
| Creatinine (µmol/L) | -0,61 | 0,00 |
| eGFR (mL/min/1.73m²) | 0,56 | 0,00 |
| mGFR (mL/min/1.73m²) | 0,61 | 0,00 |
| Proteinuria (g/L) | -0,44 | 0,02 |
| Albuminuria (g/L) | -0,47 | 0,01 |
| Creatininuria (mmol/L) | 0,37 | 0,06 |
| UCrCl (mL/min) | 0,41 | 0,04 |

**Table S4.** Residuals between mGFR and eGFR (mGFR - eGFR) by time-point, mL/min per 1.73m2. RMSE: Root Mean Square Error, BSA: body surface area

| **Period** | **Equation** | **Median**  **[IQR]** | **Mean**  **[IC95%]** | **RMSE** |
| --- | --- | --- | --- | --- |
|  | | | |  |
| Pre-Cystectomy | BSA adjusted Cockcroft-Gault | -9.4  [-25.4 ; - 0.1] | -14.4  [-22.3 ; -6.5] | 20.0 |
|  | MDRD | -14.9  [-29.7 ; -4.8] | -17.5  [-24.2 ; -10.8] | 16.9 |
|  | CKD-EPI | -4.6  [-16.4 ; 2.8] | -8.5  [-13.7 ; -3.2] | 12.4 |
|  | BSA adjusted Janowitz | -2.8  [-20.2 ; 7.7] | -5.3  [-11.2 ; 0.7] | 8.2 |
|  | FAS | -7.9  [-20.9 ; 1.5] | -10.2  [-16.9 ; -3.5] | 17.2 |
|  | | | |  |
| Post-Cystectomy | BSA adjusted Cockcroft-Gault | -12.1  [-17.3 ; -4.3] | -11.6  [-16.3 ; -7.0] | 11.8 |
|  | MDRD | -12.1  [-17.5 ; -5.4] | -14.7  [-20.3 ; -9.1] | 13.9 |
|  | CKD-EPI | -12.0  [-18.1 ; -3.5] | -12.0  [-15.2 ; -8.8] | 8.0 |
|  | BSA adjusted Janowitz | -8.9  [-17.6 ; -7.0] | -12.1  [-16.9 ; -7.8] | 5.5 |
|  | FAS | -11.0  [-17.8 ; 1.5] | -9.7  [-14.6 ; 4.8] | 12.6 |
